# Exploratory Analysis of Plasma Angiotensin-Converting Enzyme 2 and Angiotensin Peptides as Candidate Discriminatory Signals in Breast Cancer: A Pilot Case–Control Study

**DOI:** 10.3390/biomedicines14051086

**Published:** 2026-05-12

**Authors:** Biwash Ghimire, Pradeep Giri, Susan Tavernier, Sarah E. Hobdey, Ali Aghazadeh-Habashi

**Affiliations:** 1 College of Pharmacy, Idaho State University, Pocatello, ID 83209, USA; biwashghimire@isu.edu (B.G.); pradeepgiri@isu.edu (P.G.); 2 School of Nursing, University of Alaska Anchorage, Anchorage, AK 99508, USA; sstavernier@alaska.edu; 3 Department of Biomedical and Pharmaceutical Sciences, Idaho State University, Meridian, ID 83642, USA; sarahhobdey@isu.edu; 4 Veterans Affairs Medical Center, Boise, ID 83702, USA; 5 Idaho Veterans Research and Education Foundation, Boise, ID 83724, USA

**Keywords:** ACE2, angiotensin peptides, cytokines, LC-MS/MS, multi-analyte model, breast cancer biomarkers

## Abstract

**Background:** The renin-angiotensin system (RAS), traditionally known for its role in cardiovascular regulation, has also emerged as a key regulator of tumor progression and metastasis. Dysregulation of the RAS components has been implicated in breast cancer due to the significant presence of the RAS-related proteins in the breast tissue. This study aims to identify the dysregulated RAS components and investigate their potential as candidate biomarkers. **Methods**: A pilot case–control study was carried out with 21 treatment-naïve breast cancer patients and 17 healthy controls. Plasma levels of Ang 1-7, Ang II, ACE2 and selected cytokines (IL-6, IL-8, IL-10 and IFN-γ) were measured using LC-MS/MS and ELISA. ROC curves were used to assess changes in biomarker levels across the RAS components. **Results**: This pilot cohort showed evidence of altered circulating RAS-related analytes and IL-10 in treatment-naïve breast cancer patients compared with controls. The ratio of Ang 1-7/Ang II was reduced by over two-fold in breast cancer patients (*p* = 0.0442). While plasma ACE2 was significantly elevated in breast cancer patients (*p* = 0.0005), IL-10 was significantly suppressed (*p* = 0.0420). In exploratory logistic regression analysis, ACE2 showed potential as a classifier with improved discrimination when combined with Ang 1-7 and Ang II (AUC = 0.9396 [95% bootstrap CI: 0.84–1.00], accuracy = 92.59% at the Youden-optimized threshold). However, due to the small sample size and methodological limitations, these findings require further validation. **Conclusions**: In this exploratory pilot study, plasma ACE2, the Ang 1-7/Ang II ratio, and IL-10 showed promising discriminatory performance. However, these findings are hypothesis-generating and require external validation in larger, prospectively enrolled cohorts before any clinical inference can be drawn.

## 1. Introduction

Breast cancer is the most prevalent cancer among women, affecting millions of people around the world, characterized by its heterogeneous nature and variable outcomes. Due to the complex nature of the pathophysiology of breast cancer, it is essential to understand the molecular mechanisms responsible for tumor development and advancement. Among these mechanisms, the Renin-Angiotensin System (RAS) has received significant attention for its role in cancer biology. RAS is primarily known for its functions in cardiovascular regulation and fluid balance. However, recent studies have also explored its role in various cancer processes, including cell proliferation, migration, and angiogenesis [1,2,3].

The RAS is a complex cascade of peptides, enzymes, and receptor proteins composed of two distinct and opposing arms. The precursor protein of the RAS, Angiotensinogen, is cleaved by Renin to release the decapeptide Angiotensin I (Ang I) [4]. Ang I is then cleaved by the angiotensin-converting enzyme (ACE) to form the biologically active peptide Angiotensin II (Ang II). Ang II modulates a diverse range of physiological effects, including vasoconstriction, vascular smooth muscle cell proliferation, and hypertrophy of the heart vessel wall, through its interaction with the Ang II type 1 receptor (AT1R) [5]. The axis of Ang II/ACE/AT1R is known as the classical arm of RAS. A homolog of ACE, ACE2, cleaves Ang II to Angiotensin 1-7 (Ang 1-7). Ang 1-7 is a heptapeptide with affinity to the Mas receptor (MasR), to produce effects that directly oppose the actions of Ang II [6,7,8]. This axis of Ang 1-7/ACE2/MasR is known as a protective axis of the RAS, which acts as a counter-regulatory to the classical arm [9].

The discovery of ACE2 has revealed its role in maintaining the balance between the two RAS axes. Circulating levels of soluble ACE2 (sACE2) in plasma and urine have been linked to various diseases, including cancer [10,11,12,13,14]. The increase in plasma levels of sACE2 may be considered a part of the body’s defense mechanism to neutralize higher Ang II levels. Higher urinary or plasma levels of sACE2 were reported in patients suffering from hypertension, heart failure, microalbuminuria, and overt vascular disease [15,16].

In breast tissue, the classical RAS promotes tumorigenesis through AT1R-mediated activation of proliferative, anti-apoptotic, and pro-metastatic signaling cascades [1,2,17,18,19,20]. Conversely, the counter-regulatory axis has been shown to attenuate tumor growth, inhibit angiogenesis, and reduce metastatic potential in preclinical models [21,22,23].

The RAS is a central factor in breast cancer angiogenesis, acting through the classical pathway. Ang II is a key stimulator of vascular endothelial growth factor (VEGF), which is a driver of angiogenesis [24]. Angiogenesis is a crucial hallmark of cancer that supports supplying nutrients and oxygen to the rapidly growing tumor mass [1,14,21].

The immunomodulatory function of the RAS is well-studied and well-known. The infamous cytokine storm in COVID-19 patients has been directly linked to the overactivation of the RAS, driven by reduced lung ACE2 levels [25]. This overactivation activates nuclear factor kappa B (NF-κB) and MAPK signaling, which drive the secretion of inflammatory cytokines such as IL-1, IL-6, and TNF-α, further augmenting the inflammatory process [26,27]. IL-10 is a potent anti-inflammatory cytokine; however, its pleiotropic effects make it a paradoxical, context-dependent regulator, acting as both a pro- and anti-cancer cytokine [28]. While activation of the Ang 1-7/MasR axis suppresses these actions, it is essential to understand the complex interplay between RAS and the inflammatory cascade in breast cancer.

Despite growing evidence linking RAS dysregulation to breast cancer, no study has simultaneously quantified circulating ACE2, Ang 1-7, and Ang II in treatment naïve patients to evaluate whether a multi-analyte RAS imbalance signature can discriminate patients from healthy controls. This pilot case–control study addresses that gap by measuring plasma ACE2, Ang 1-7, Ang II and a panel of cytokines in treatment naïve breast cancer patients and healthy controls and by assessing their individual and combined discriminatory performance.

## 2. Materials and Methods

### 2.1. Study Design and Ethical Approval

The institutional review board approved the study through the protocol number IRB-FY2020-273. Pre-collected plasma samples from breast cancer patients from St. Luke’s Hospital (Boise, ID, USA) and healthy control individuals’ samples from SpeciCare (Gainesville, GA, USA) were purchased for analysis. This study was exempt from the consent requirement as it falls under Category 4 (ii) of the approved IRB protocol.

The samples were venous blood plasma collected with EDTA coagulant. The samples were preserved by freezing them at −80 °C and transported frozen on dry ice. Samples underwent one freeze–thaw cycle after receiving and before analysis. A protease inhibitor cocktail was added to all aliquots prior to peptide analysis, according to the manufacturer’s instructions.

### 2.2. Study Population and Participant Characteristics

**Figure 1:**
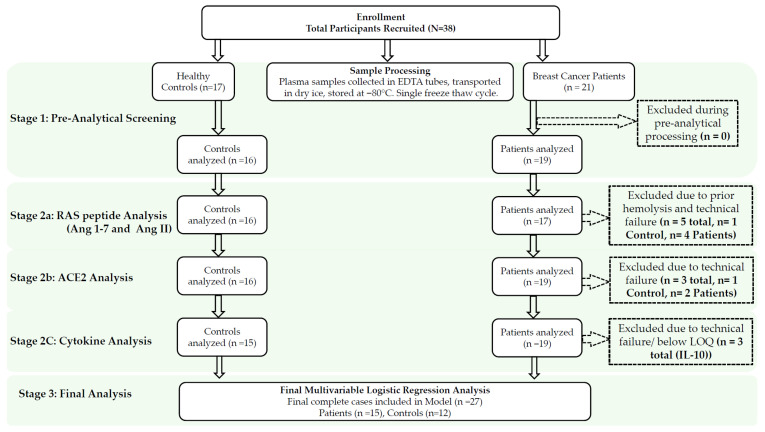
CONSORT style flowchart illustrating sample enrollment, processing, and analysis. The diagram shows the progression of the study from initial sample of *n* = 38 through technical analysis and final model inclusion. A total of 21 breast cancer patients and 17 healthy controls were enrolled. 5 samples in total including 1 control and 4 patients were excluded from the RAS peptide analysis due to hemolysis and technical failure. For the ACE2 ELISA experiment, 1 control and 2 patient samples were excluded due to their coefficient of variance between the replicated were higher than 20% threshold. For the cytokine analysis, 3 samples were omitted for IL-10 analysis, whereas the rest of the cytokine panel (IL-1α, IL-1β, IL-6, IL-8, IFN-γ, and TNF-α) had 50% of samples below LOQ. Therefore, many samples were excluded, making the finding for these 6 specific analytes unreliable.

As this study was designed as a pilot case–control study to explore the feasibility of circulating RAS components as biomarkers in treatment-naïve breast cancer, the sample size (*n* = 38) was determined based on the availability of clinically well-characterized treatment-naïve patients and the exploratory nature of the investigation. All breast cancer patients were treatment-naïve, defined as none of the patients being subjected to chemotherapy or radiotherapy prior to sample collection. Figure 1 demonstrates the Consolidated Standards of Reporting Trials (CONSORT) style flowchart summarizing the number of samples used in the analysis. Laboratory personnel performing analysis were not blinded to case/control group assignments.

### 2.3. Inclusion and Exclusion Criteria

The inclusion criteria for the breast cancer patients were treatment-naïve female cancer patients aged over 18 years. Inclusion criteria for control groups were similar-aged female volunteers with no history of cancer. No exclusion criteria were applied to either cohort. No stratification by tumor stage or receptor status was performed a priori.

### 2.4. Materials

Ang 1-7 (Anaspec, AS-61039) and Ang II (Anaspec, AS-20633) were purchased from Anaspec Inc. (Fremont, CA, USA). The internal standard (IS) (Asn1, Val5)-Ang II (Sigma-Aldrich A6402-1MG) was purchased from Sigma-Aldrich (St. Louis, MO, USA). cOmplete™ Mini Protease Inhibitor Cocktail (Sigma-Aldrich, St. Louis, MO, USA) is used to stabilize plasma samples by preventing protein and peptide degradation. Waters C18 SPE cartridges (Sep-Pak WAT020805) were obtained from Waters (Milford, MA, USA). LC-MS-grade solvents, including water, acetonitrile, and formic acid, were procured from Fisher Scientific (Fair Lawn, NJ, USA). A positive-pressure manifold for solid-phase extraction was bought from Agilent Technologies (Agilent Technologies Inc., Santa Clara, CA, USA). Human ACE2 ELISA kit was acquired from RayBiotech (Peachtree Corners, GA, USA), and custom Procartaplex 7-plex plate was received from Life Technologies Corporation (Grand Island, NY, USA).

### 2.5. LC-MS/MS System

The utilized LC-MS/MS system comprised liquid chromatography system purchased from Shimadzu Scientific Instruments, Columbia, MD, USA, consist of a binary pump (LC-30AD), an autosampler (SIL-30AC), a controller (CBM-20A), a degasser (DGU-20A5R), a column oven (CTO-20A), and an ABSciex QTRAP 5500 mass spectrometer (SCIEX, Foster City, CA, USA) with an electron spray ionization (ESI) source. The Analyst 1.7 software (SCIEX, Foster City, CA, USA) was used to monitor and analyze chromatograms. The sample separation was accomplished using a Synergi™ Fusion-RP column (2.5 µm, 100 × 2 mm) obtained from Phenomenex (Torrance, CA, USA). All analyses were carried out in positive-ion mode. 

### 2.6. Sample Preparation Procedures

Ang 1-7 and Ang II were extracted from human plasma using a Solid Phase Extraction (SPE) technique based on a previous method reported by Cui et al., with slight changes [29,30]. 200 µL of the plasma was mixed with 100 µL of 25 ng/mL IS. Then, followed by adding 2 µL of formic acid to achieve the final concentration of 1%. SPE cartridges were preconditioned with 3 mL of ethanol and 3 mL of deionized water in a series. Plasma samples pre-mixed with a protease inhibitor cocktail were loaded onto the cartridge and allowed to interact with the stationary phase, followed by application of positive nitrogen flow via a positive-pressure manifold. The column was then washed with 3 mL of deionized water, followed by elution of the peptides of interest with 3 mL of methanol containing 5% formic acid. The eluent was collected and dried using a SpeedVac system (Thermo Fisher Scientific, Waltham, MA, USA). The dried samples were reconstituted in 100 µL of water containing 0.1% formic acid. An aliquot of 30 µL was injected into the LC-MS/MS system to quantify angiotensin peptide concentrations.

### 2.7. LC-MS/MS Method

Ang 1-7, Ang II and IS were eluted at different retention times using a Synergi RP column (C-18, 100 × 2 mm ID) with a particle size of 2.5 µm obtained from Phenomenex (Torrance, CA, USA). The column temperature was set at 40 °C. The mobile phase was composed of 0.1% formic acid in water (mobile phase A) and 0.1% formic acid in acetonitrile (mobile phase B), pumped as a gradient elution. The gradient program was established as 5% ACN to 30% ACN for 4 min. The gradient was held constant for 4–8 min, then reduced to 5% ACN at 9 min and maintained for another 1 min. The flow rate was set at 0.3 mL/min, and the injection volume was 30 µL.

The analytes were detected in positive ion mode using multiple reaction monitoring (MRM) in an Electrospray ionization (ESI) source. Previously optimized source gas parameters were used [30]. The set parameters are as follows: curtain gas, 30 psi; collision gas, medium; ion-spray voltage, 5500 V; temperature, 300 °C; nebulizer gas (ion source gas 1), 20 psi; and turbo gas (ion source gas 2), 25 psi. The specific MRM transition of m/z values used for quantification of Ang 1-7 were 450.4 → 647.0 and 450.4 → 110.1. Ang II was analyzed using m/z of 523.9 → 263.3 and 523.9 → 784.5. (Asn1, Val5)-Ang II used as internal standard was analyzed at m/z of 516.5 → 769.4 and 516.6 → 110.1. Collision energy (CE) and declustering potential (DP) used for Ang 1-7 were 20 and 90 V, for Ang II were 30 and 25 V, and for IS were 25 and 25 V, respectively. An entrance potential of 10 V and a cell exit potential (CXP) of 20 V were used for all the analytes.

### 2.8. Measurement of Plasma *ACE2*

Plasma levels of ACE2 were measured by enzyme-linked immunosorbent assay (ELISA) with the Human ACE2 ELISA kit (RayBiotech, Peachtree Corners, GA, USA) in duplicate, according to the manufacturer’s recommendations. Briefly, 100 µL of the standard solutions and samples were applied to the commercial ELISA plate pre-coated with capture antibodies and incubated for 2.5 h at room temperature. The plate was washed with the washing solution, and 100 µL of biotin antibody was added to each well, which was incubated for 1 h. The plate was washed again, then the Streptavidin solution was added and incubated for 45 min. For colorimetric detection, after adding 100 µL of TMB substrate, the reaction was incubated for 30 min, then stopped by adding 50 µL of stop solution. The absorbance was measured immediately at 450 nm.

### 2.9. Measurement of Cytokine Levels

An Immune-bead array assay was performed using bead-based technology with a custom Procartaplex 7-plex (Life Technologies Corporation, Grand Island, NY, USA) and analyzed on the Luminex platform (Luminex Corporation, Austin, TX, USA). 25 μL plasma samples were diluted 4-fold, then aliquoted into the 96-well preconfigured plate and analyzed for interleukins (IL)-1, IL-6, IL-8, IL-10, IFN-γ, and TNF-α according to the manufacturer’s instructions. Standards were run alongside the samples, prepared in duplicate.

### 2.10. Statistical Analysis

The data were presented as means ± standard error of mean (SEM) unless stated otherwise. They were tested for normality using the Shapiro–Wilk test. A group comparison of normally distributed data was performed using Student’s *t*-test with Welch correction assuming unequal variances. In contrast, non-normal data were analyzed using the Mann–Whitney U test. The correlation of continuous variables was quantified using Spearman’s correlation coefficient. For Mann–Whitney U group comparisons, effect size is reported as rank-biserial correlation (r), where r = 0.1 indicates a small effect, r = 0.3 a medium effect, and r = 0.5 a large effect. For *t*-tests, effect size is reported as Cohen’s D (D), where D = 0.2 indicates a small effect, D = 0.5 a medium effect and D = 0.8 a large effect. The predictive capacity of the variables was initially assessed using univariate logistic regression for each predictor, followed by multivariable models incorporating additional variables. In these analyses, the response variable was binary, representing the presence or absence of breast cancer. The strength of association was determined using the odds ratio (OR), and the discriminatory performance of the models was assessed using receiver operating characteristic (ROC) analysis. The confidence interval level was set at 95%, and *p* ≤ 0.05 was considered statistically significant. Given the exploratory nature of this pilot study, no correction for multiple testing was applied in primary analyses. All *p*-values should be interpreted in the context of multiple comparisons, and the increased risk of type I error is acknowledged as a study limitation. Findings are intended to generate, rather than confirm, hypotheses for future hypothesis-driven studies.

Internal validation was performed using bootstrap resampling with 1000 iterations. For each bootstrap sample, data were resampled with replacement, and the logistic regression model (ACE2 + Ang 1-7 + Ang II) was refitted. Model performance was assessed by calculating the optimism-corrected AUC. Optimism was defined as the difference between the apparent AUC (from the original dataset) and the mean bootstrap AUC. The optimism-corrected AUC represents the expected performance in new data. Bootstrap 95% confidence intervals were calculated using the percentile method (2.5th and 97.5th percentiles of the bootstrap distribution).

Given the small sample size and ACE2’s strong discriminatory performance, quasi-complete separation was anticipated in the standard logistic regression model. To obtain stable, bias-corrected estimates, Firth’s penalized logistic regression was applied as a supplementary analysis [31]. The univariate Firth model was fitted on all participants with available ACE2 measurements (*n* = 35), while the full multivariable Firth model was fitted on the complete-case subset with all three predictors available (*n* = 27; 13 controls, 14 patients). AUC was calculated from predicted probabilities of each Firth model using ROC analysis.

Statistical analysis of group comparisons and correlations, along with their visualization, was performed in GraphPad Prism 10. Logistic regression, stepwise selection for identifying an optimal set of variables, and bootstrap resampling were performed in Jamovi 2.6.26. Post hoc power analysis was performed in Gpower 3.1.9.7 to provide insight into the required sample sizes for future studies. Data visualization for logistic regression and logit identification was performed in R version 4.5.0, RStudio 2025.05.0 build 496. The list of libraries used in R analyses is as follows: tidyverse, readxl, ggplot2, ggpubr, dplyr, pROC, forestplot, broom, pROC and gridExtra [32,33,34,35,36].

## 3. Results

### 3.1. Characteristics of Control and Patient Cohort

Our study analyzed de-identified plasma samples from 17 healthy subjects and 21 breast cancer patients having different tumor grades at different clinical stages. The variables such as age, body mass index (BMI), medication history, comorbidities and various clinical stages of breast cancer were considered for further analysis with respect to their association with the RAS peptides.

The study cohort consisted exclusively of women in both the control and patient groups. Although control and healthy subjects were not matched, the average ages of the healthy and patient populations were 60.90 ± 12.70 years and 65.14 ± 12.98 years, respectively (*p* = 0.3208), suggesting the groups were comparable in age. The mean BMI of the patients was in the class I obesity range at 33.44 ± 14.60 kg/m^2^.

The clinical stages of these patients were based on the standard pTNM classification, which uses TNM (tumor, node, and metastasis) staging systems based on the excised or physically examined tumor to define the extent of cancer. In this classification, pT1 refers to tumors measuring 1–2 cm, whereas pT2 refers to tumors measuring 2–5 cm across. pT1 is subdivided into pT1mi (<0.1 cm), pT1a (0.1–0.5 cm), pT1b (0.5–1 cm), and pT1c (1–2 cm). Most tumors (*n* = 17, 80.95%) were pT1, indicating a tumor size less than 2 cm at its greatest dimension. This group was subdivided into pT1a (*n* = 4, 19.05%), pT1b (*n* = 4, 19.05%), and pT1c (*n* = 9, 42.85%). The remaining patients’ tumors were pT2 (*n* = 3, 14.29%), suggesting that the majority of participants had tumors measuring 1–2 cm (Table 1).

Pathological N categories (pN classification) describe the extent of lymph node involvement by cancer. Here, pN0 means no regional lymph node metastasis was identified (*n* = 16, 76.19%), pN1 (*n* = 4, 19.05%) suggests the cancer has spread to 1–3 axillary lymph nodes. pN1 is further divided into pN1mi (*n* = 1, 4.76%) referring to micro-metastasis of cancer cells between the size of 0.2–2 mm, pN1a (*n* = 2, 9.52%) in which cancer has spread to 1–3 axillary lymph nodes with at least one metastasis exceeding 2 mm. pN1b category suggests cancer is present in internal mammary lymph nodes on the same side as the primary tumor. When both pN1a and pN1b criteria are met, it is designated as pN1c (*n* = 1, 4.76%) [37,38].

Tumor grades are based on how closely the cancer cells resemble normal cells of the tissue. Most participants had a grade 1 tumor (G1; *n* = 16, 76.19%), indicating that the cancer cells are slow-growing and well-differentiated, with a strong resemblance to healthy cells. Grade 2 (G2; *n* = 3, 14.29%) suggests moderately differentiated, faster-growing cells, whereas grade 3 (G3; *n* = 1, 4.76%) consists of very aggressive, poorly differentiated cancer cells (Table 1) [39]. Depending on the level of human epidermal growth factor receptor 2 (HER2) expression in breast cancer cells, they are classified as HER2-positive (HER2^+^) or HER2-negative (HER2^−^) [40]. HER2^+^ cancers are considered more aggressive as the HER2 protein is involved in mitotic signaling, leading to faster growth [41]. Our study cohort included 2 patients (9.52%) with HER2^+^ cancer. Similarly, based on estrogen receptor (ER) and progesterone receptor (PgR) status, breast cancer is divided into ER^+^/ER^−^ and PgR^+^/PgR^−^ groups. Our study population included *n* = 18 (85.71%) ER^+^ and *n* = 17 (80.95%) PgR^+^ (Table 1).

Study participants reported cardiovascular and cardiometabolic comorbidities, including Diabetes (*n* = 2, 9.52%), Cardiac arrhythmia (*n* = 4, 19.05%), hypertension (*n* = 10, 47.62%), peripheral vascular disease (*n* = 1, 4.76%), and congestive heart failure (*n* = 1, 4.76%) (Table 1). The participants also reported use of Aspirin at any dose (*n* = 3, 15%), non-steroidal anti-inflammatory drugs (NSAIDs) (*n* = 4, 19.05%), and statins (*n* = 4, 19.05%). 47.62% (*n* = 10) of the patients reported using tobacco in any form. Specifics regarding the status, duration and frequency of tobacco use were not available. The average value of serological markers was within the normal range (Table 1).

### 3.2. Effect of Breast Cancer on *Ang 1-7* and *Ang II* Levels

The Ang 1-7 and Ang II concentrations were calculated by comparing the areas of the peaks of the analytes obtained from the plasma sample to the standard curve drawn from spiked blank plasma in the LC-MS/MS chromatograms. The method was previously developed and validated in accordance with the International Council for Harmonization (ICH) guidelines. Briefly, the analytical method validation included intra-day and inter-day precision analysis for concentrations ranging from 0.78–100 ng/mL for Ang 1-7, and 0.05–100 ng/mL for Ang II, with an acceptable coefficient of variance (CV < 15.49%). The concentration ranges showed a linear response with a linearity factor (r^2^) greater than 0.99 and a matrix effect within an acceptable range of 90.02–106.81%. The lower limit of quantification (LLOQ) was determined to be 0.25 ng/mL for Ang 1-7 and Ang II.

Among the samples analyzed, the resulting Ang 1-7 and Ang II data for two patient samples and one control sample were omitted because the plasma samples were hemolyzed. They were, however, considered for the ratio as they were indicative of RAS balance. Two samples, one from each of the Ang II control and patient groups, were excluded from analysis because they were below the limit of quantification. Two samples from the control group were omitted due to persistent column blockage during the analysis. The final sample sizes in the Ang 1-7 groups were 16 and 17; the Ang II groups were 14 and 15; and the ratios were 15 and 17 for the control and patient groups, respectively (Figure 1).

**Figure 2:**
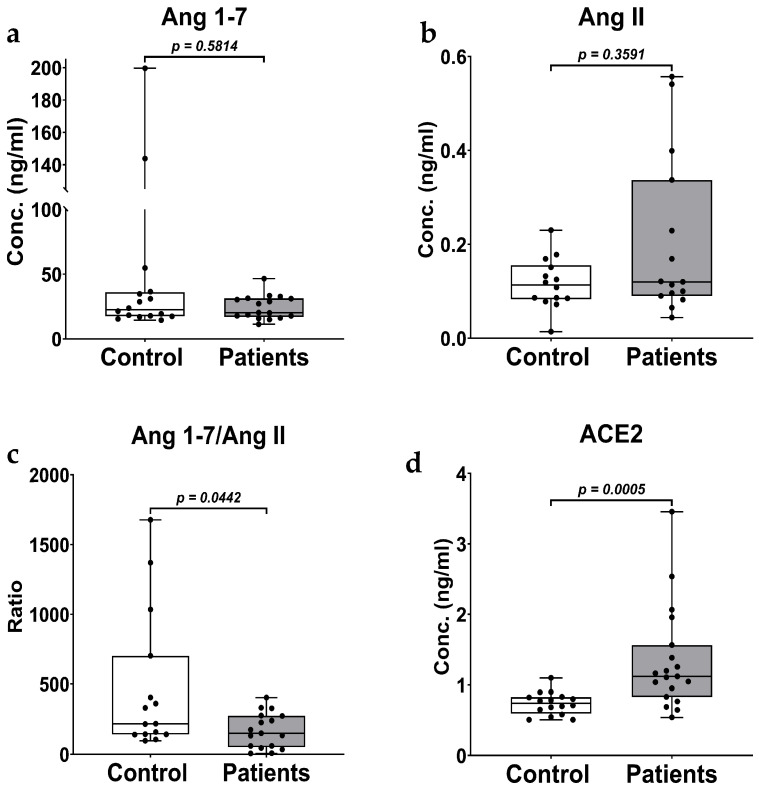
The boxplots overlayed with scatterplots of plasma concentrations of Ang 1-7 (**a**), Ang II (**b**), and the Ang 1-7/Ang II ratio (**c**) in the control group (*n* = 16) and breast cancer patients (*n* = 17). The boxplot of plasma ACE2 concentration (**d**) in the control group (*n* = 17) and breast cancer patients (*n* = 19). Statistical comparisons were performed using the Mann–Whitney U test; statistical significance was set at *p* < 0.05.

We did not see a significant difference (rank-biserial, r = −0.1177, *p* = 0.5814) between plasma levels of Ang 1-7 between the control (43.40 ± 13.04 ng/mL) and the breast cancer patients (24.39 ± 2.23 ng/mL) (Figure 2a). Plasma levels of Ang II were elevated in breast cancer patients (0.20 ± 0.04 ng/mL) compared to the controls (0.12 ± 0.01 ng/mL), but not significantly different (rank-biserial, r = 0.2048, *p* = 0.3591) (Figure 2b). To assess the balance of the RAS effector peptides between the two groups, Ang 1-7 levels were normalized to Ang II levels. The resulting ratio was significantly lower (rank-biserial, r = −0.4196, *p* = 0.0442) in breast cancer patients (169.47 ± 30.33) than in controls (473.20 ± 129.07) (Figure 2c), suggesting a significant RAS pathway dysregulation in breast cancer patients. 

### 3.3. Effect of Breast Cancer on Plasma *ACE2*

The concentrations of ACE2 in the plasma samples were determined by comparing the average absorbance values of duplicate samples to a standard curve generated from the standards. A standard curve was drawn over a concentration range of 0.027 ng/mL to 20 ng/mL. The LLOQ for ACE2 was 0.025 ng/mL according to the kit manufacturer’s data. Two patients’ samples with a coefficient of variance (CV) greater than 20% were omitted for analysis. One sample from the patient group was considered a technical failure because its value was over 200 times the average of the remaining samples (Figure 1).

Plasma ACE2 in the breast cancer patients (1.33 ± 0.17 ng/mL) was significantly higher (rank-biserial, r = 0.6678, *p* = 0.0005) than the levels in the control population (0.74 ± 0.04 ng/mL) (Figure 2d).

### 3.4. Effect of Breast Cancer on Cytokine Levels

**Figure 3:**
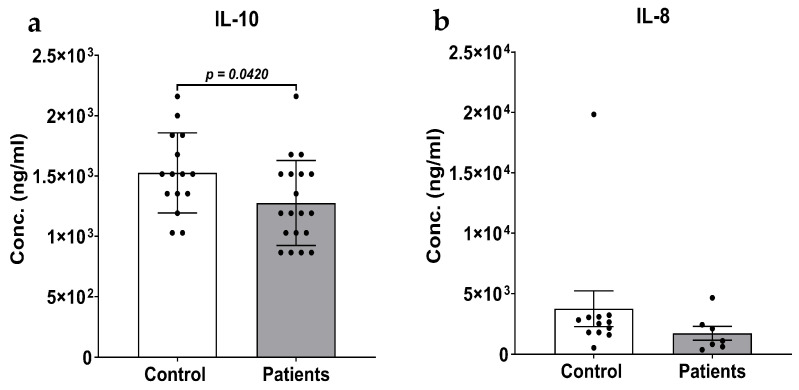
Bar graphs with scatter plots of plasma concentrations of IL-10 (**a**), and IL-8 (**b**) in the control group and in breast cancer patients. Data are presented as mean ± SEM. Statistical comparison between the groups (Controls, *n* = 15 and Patients, *n* = 19) was performed using Student’s *t*-test for IL-10, and statistical significance was set at *p* < 0.05. No group comparison was carried out for IL-8 (*n* = 12, 7) due to at least one group having more than 50% samples below limit of quantification.

There was a significant reduction (*p* = 0.0420, D = 0.7302, 95% CI = 0.0096, 0.4896, *n* = 15, 19) in plasma IL-10 levels in patients (1.28 ± 0.08 pg/mL) compared to controls (1.53 ± 0.09 pg/mL) (Figure 3a). Data below the limit of quantification (LOQ) were omitted from analysis, which included two samples each from the control and patient groups for IL-10 (LOQ < 0.8 pg/mL).

More than 50% of samples in at least one group for IL-6, IL-8 (Figure 3b), and IFN-γ fell below the limit of quantification (IL-6: LOQ < 2.0 pg/mL; IL-8: LOQ < 0.3 pg/mL; IFN-γ: LOQ < 7 pg/mL). Descriptive statistics for these analytes are provided in Supplementary Material Table S1,and should be interpreted as hypothesis-generating only. The plasma concentrations of TNF-α, IL-1α, and IL-1β were below the detection limit for all samples and were not reported further.

### 3.5. Exploratory Sensitivity Analysis for Comorbidity as a Confounding Variable

Due to the high prevalence of hypertension (47.62%) and tobacco use (47.62%) in the patient cohort, exploratory sensitivity analyses were conducted to evaluate potential confounding on primary outcomes. Full results are provided in the Supplementary Materials under “Exploratory Subgroup Analyses”. Briefly, plasma ACE2 remained significantly elevated (*p* < 0.0001) in non-hypertensive cancer patients (1.65 ± 0.62 ng/mL) compared to the control cohort (0.74 ± 0.04 ng/mL), suggesting that the primary ACE2 finding is not solely attributable to hypertension-related confounding. However, the subgroup sample sizes (*n* ≤ 10/subgroup) are below the threshold for reliable estimation. Therefore, these findings should be interpreted as exploratory only. ACE2 was also significantly lower in the hypertensive cancer patient cohort compared to non-hypertensive cancer patients (rank-biserial r = −0.7778, *p* = 0.0120), consistent with prior literature, although the estimate was unreliable due to a small sample size and co-occurring cardiovascular comorbidities.

### 3.6. Correlation of RAS Components and *IL-10*

Plasma concentration of Ang 1-7 did not show any correlation with the plasma concentration of Ang II (r = 0.1641, *p* = 0.3950), suggesting the possibility that Ang II may not be a primary substrate for the formation of Ang 1-7 (Supplementary Material Table S2). A similar finding was reported in a previous study of hypertensive subjects, in which these two effector peptides were negatively correlated in males but showed no correlation in females [42]. Ang 1-7 did not show significant correlations with ACE2 (r = −0.3322, *p* = 0.0729) or IL-10 (r = 0.3236, *p* = 0.0811) (Supplementary Material Table S2). Similarly, Ang II was not correlated with ACE2 (r = 0.1811, *p* = 0.3659) or IL-10 (r = −0.1092, *p* = 0.5954) (Supplementary Material Table S2). ACE2 also showed a moderately negative association with IL-10 (r = −0.4123, *p* = 0.0212).

### 3.7. Correlation of RAS Components and *IL-10* with Biological Variables

**Figure 4:**
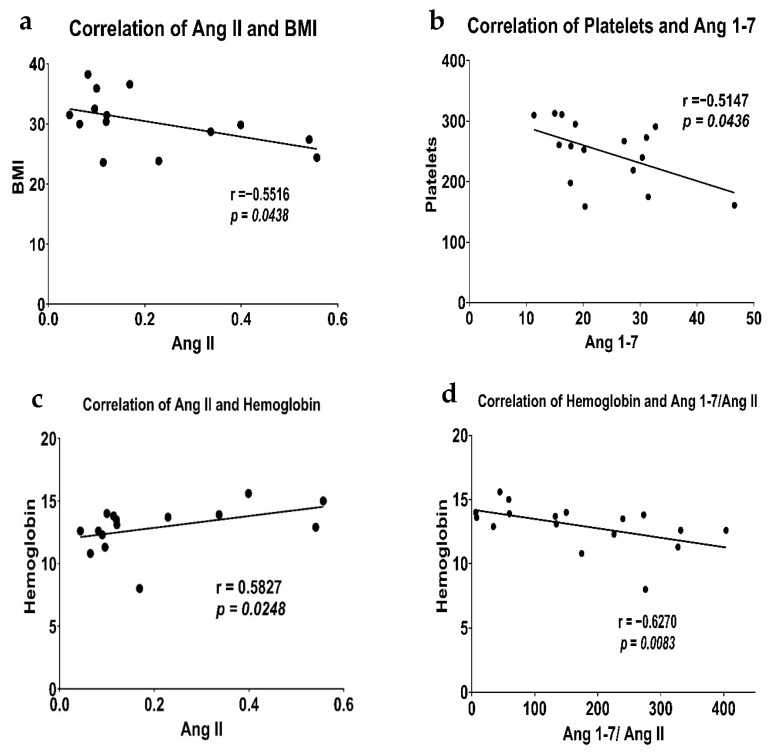
The correlation graphs of plasma concentrations (ng/mL) of Ang II against BMI (kg/m^2^) (**a**), Ang 1-7 (ng/mL) against platelets (counts per µL) (**b**), Ang II (ng/mL) against Hemoglobin (g/dL) (**c**), and Ang 1-7/Ang II ratio against hemoglobin (g/dL) (**d**). A statistical test was performed using Spearman’s correlation coefficient (r), with *p* < 0.05 considered statistically significant.

The biological variables used in this study are age and BMI. The age data were available for both the control and patient groups, but the BMI data were available only for the patient group. Therefore, the correlation between BMI and analytes was not performed in the control group. There was no significant correlation of age with Ang 1-7 (r = 0.0982, *p* = 0.5865), Ang II (r = 0.2076, *p* = 0.2798), Ang 1-7/Ang II (r = −0.2611, *p* = 0.1498), ACE2 (r = 0.0960, *p* = 0.5833), or IL-10 (r = 0.0449, *p* = 0.8009) (Supplementary Material Table S2). While BMI showed strong correlation with Ang II (r = −0.5516, *p* = 0.0438) in the breast cancer patients (Figure 4a, Supplementary Material Table S2), it did not show any correlation with Ang 1-7 (r = −0.0676, *p* = 0.8051), Ang 1-7/Ang II (r = 0.2426, *p* = 0.3467), ACE2 (r = 0.1084, *p* = 0.6687), or IL-10 (r = 0.2043, *p* = 0.4161) (Supplementary Material Table S2).

### 3.8. Correlation of RAS Components and *IL-10* with Hematological and Biochemical Markers

Hematological markers, comprising quantified hemoglobin, white blood cells (WBC), and Platelets, along with biochemical markers including serum bilirubin and creatinine, were correlated with the RAS components and IL-10 of breast cancer patients to understand how dysregulation of the RAS impacts these variables. Ang 1-7 showed a strong negative correlation with platelets (r = −0.5147, *p* = 0.0436) (Figure 4b, Supplementary Material Table S2). Hemoglobin was positively correlated with Ang II (r = 0.5827, *p* = 0.0248), whereas it was negatively correlated with Ang 1-7/Ang II ratio (r = −0.6270, *p* = 0.0083) (Figure 4c,d). Similarly, ACE2 showed a strong correlation with WBC (r = 0.5515, *p* = 0.0237). IL-10 did not show a significant correlation with any of the above-mentioned serological markers. We did not see any correlation between serum bilirubin and creatinine and the RAS peptides or IL-10 levels. The complete correlation matrix between the groups is presented in Supplementary Material Tables S2 and S3.

### 3.9. Logistic Regression

A binomial logistic regression was performed to identify variables associated with the likelihood that individuals in the study population would be in the Control or Patient group. We developed a model via bidirectional stepwise regression. Initially, we used a univariate baseline, creating a separate model for each variable (Ang 1-7, Ang II, Ang 1-7/Ang II, ACE2, and IL-10) with our groups (Controls and Patients) as the dependent variables. The performance of the model was assessed using Akaike Information Criterion (AIC), deviance, McFadden’s pseudo R^2^ (R^2^_McF_), and the *p*-value of the predictor. In the univariate model, ACE2 was determined as the best predictor (AIC = 36.49, deviance = 32.49, R^2^_McF_ = 0.3269, and *p* < 0.0001).

After determining ACE2 as a baseline predictor, we added each of the remaining variables (Ang 1-7, Ang II, Ang 1-7/Ang II, and IL-10) to generate bivariate models. The model assessment was conducted using the same metrics as before, with the addition of the variance inflation factor (VIF) to account for multicollinearity-induced inflation of regression coefficients. The combination of ACE2 and Ang II was the best predictor of the disease outcome (AIC = 23.48, deviance = 17.47, R^2^_McF_ = 0.5326, and VIF = 1.05). The model consisting of ACE2 only as a predictor was defined using the following equation:

$$Logit\left(P\right)=-4.95+5.68\times [ACE2]$$

**Figure 5:**
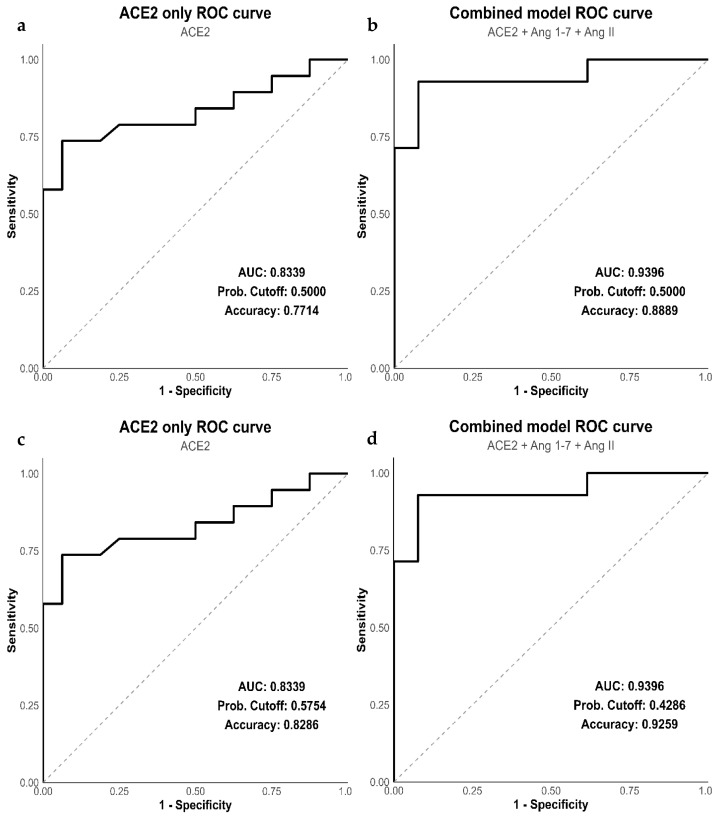
Receiver operating characteristic (ROC) curves showing the performance of the ACE2 univariate model (**a**) and the multivariate model using ACE2, Ang 1-7, and Ang II together (**b**) at an arbitrary threshold of 0.5. The ROC curves with calculated Youden’s index (J) for the ACE2 univariate model (**c**) and the multivariate model using ACE2, Ang 1-7 and Ang II together (**d**). The dashed diagonal line represents the line of no discrimination (random classifier; AUC = 0.5). ACE2, Angiotensin Converting Enzyme; Ang, Angiotensin; AUC, Area under curve; Youden’s index (J) represented as cutoff.

This forward stepwise selection process continued by adding the remaining variables sequentially based on model performance. Our optimal model was determined to be a combination of ACE2, Ang 1-7, and Ang II, with a strong model fit (R^2^_McF_ = 0.5366), AIC = 25.33, deviance = 17.33, no multicollinearity (max VIF = 1.08), and ACE2 as the significant predictor (*p* < 0.0001). Validation of this model was performed by calculating statistical performance metrics based on receiver operating characteristic (ROC) analysis, using a cutoff value of 0.5 (Figure 5a). The area under the ROC curve (AUC) was 0.9396, with an accuracy of 0.8889 (Figure 5b). This shows that our model achieved outstanding discrimination between the outcomes, with an overall accuracy of 88.89%.

Our next step was to identify the optimal threshold to maximize diagnostic accuracy. This was done by calculating Youden’s index, which maximizes the balance between sensitivity and specificity. We calculated the cutoff value (J) for our biomarker panel to be 0.4286, which was lower than the threshold for ACE2 alone (J = 0.5754) (Figure 5c). However, the combined panel of RAS biomarkers showed improved accuracy of 92.59% with a lower cutoff than 88.89% at an arbitrary cutoff of 0.5 (Figure 5d). The combined model was defined using the following equation:

$$Logit\left(P\right)=-9.64+9.57\times \left[ACE2\right]-0.007\times \left[Ang\;1-7\right]+6.3\times [Ang\;II]$$

The ROC curve showed a sharp rise in sensitivity, suggesting good group separation in this exploratory sample. The high AUC value (0.9396; 95% bootstrap CI: 0.84–1.00) must be interpreted with caution. In small datasets (*n* = 27 for the complete case multivariable model), internal validation reduces but does not eliminate optimism bias, and external validation in an independent cohort is required before any clinical inference can be drawn. Our results indicate that this panel of biomarkers (ACE2, Ang 1-7, and Ang II) demonstrated promising exploratory discriminatory performance.

**Figure 6:**
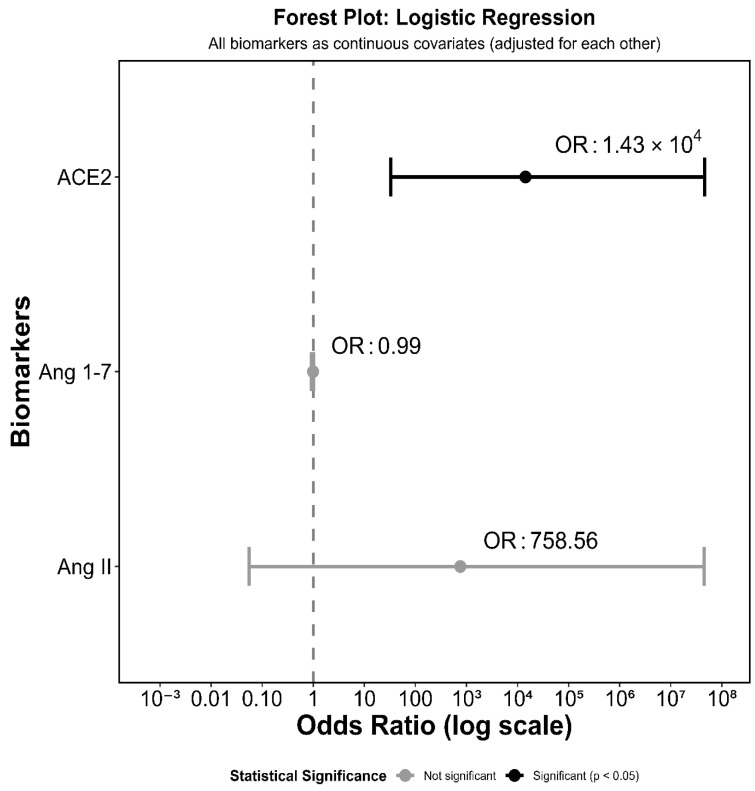
Forest plot of multivariable logistic regression showing the odds ratios (OR) and 95% CI for angiotensin converting enzyme (ACE2), Angiotensin 1-7 (Ang 1-7) and Ang II with all biomarkers treated as continuous covariates adjusted for each other. The OR is presented on a logarithmic scale. Black dots and lines represent statistically significant associations (*p* < 0.05), and gray-scale circles and lines represent non-significant associations. The vertical dashed lines represent OR of 1 corresponding to no effect.

We further calculated the odds of the outcome for each predictor variable cluster in our model. The odds ratio (OR) for ACE2 was 14282.76 (*p* = 0.0168, 95% CI = 5.59–3.65 × 10^7^), indicating that a one-unit increase in ACE2 increased the odds of breast cancer by several thousand-fold (Figure 6). Similarly, we observed a large OR (568.57) for Ang II, which was not statistically significant (*p* = 0.2561, 95% CI = 0.01–3.23 × 10^7^). Although Ang 1-7 showed minimal association with the outcome (OR = 0.99, *p* = 0.7312, 95% CI = 0.95–1.03) independently, it was an integral part of the overall multivariable model, improving model accuracy when used with ACE2 and Ang II. The extremely wide confidence intervals observed for ACE2 and Ang II indicate high variability in biomarker concentrations. These extremely wide confidence intervals for ACE2 and Ang II reflect model instability arising from small sample size and quasi-complete separation and should be interpreted accordingly. These estimates may not be suitable for clinical inference without replication.

#### 3.9.1. Bootstrap Validation

Internal validation using 1000 bootstrap resamples demonstrated minimal model optimism (optimism = −0.0103), indicating the model is not overfit to the training data. The optimism-corrected AUC was 0.9498 (95% bootstrap CI: 0.84–1.000), confirming robust discriminatory performance comparable to the apparent AUC of 0.9396. The bootstrap distribution showed consistent model performance across resamples (mean AUC = 0.9498, median = 0.9560, SD = 0.05), supporting the stability of our biomarker panel’s discriminatory ability. Some bootstrap samples exhibited quasi-complete separation due to a small sample size (*n* = 27) and strong predictor effects, leading to unstable coefficient estimates. However, AUC-based discrimination remained consistent across resamples, confirming robust model performance.

#### 3.9.2. Firth’s Penalized Logistic Regression

To address the quasi-complete separation produced by ACE2 in the standard logistic regression model, evidenced by the unstable odds ratio estimate (OR = 14,282.76, 95% CI: 5.59–3.65 × 10^7^), Firth’s penalized logistic regression was applied to obtain bias-corrected estimates. The bias-corrected estimates are tabulated in Supplementary Material Table S4. In the univariate Firth model (*n* = 35), ACE2 remained a highly significant predictor of breast cancer status (OR = 138.05, 95% CI: 4.80–14,817.63, *p* = 0.0004, AUC = 0.8339, 95% 99CI: 0.693–0.975). In the full multivariable Firth model (*n* = 27), ACE2 remained the sole significant predictor (OR = 1644.86, 95% CI: 10.29–7,910,616, *p* = 0.0004), with Ang II showing a large but non-significant positive association (OR = 49.63, 95% CI: 0.044–4,281,882, *p* = 0.271) and Ang 1-7 showing a near-neutral effect (OR = 1.00, CI: 0.9674–1.0190, *p* = 0.9902), consistent with the standard model results. The full model was statistically significant (Likelihood ratio test [LRT], *p* = 0.0018) with an AUC of 0.9339 (95% CI: 0.835–1.000), demonstrating that discriminatory performance was preserved under penalized estimation and was not attributable to separation artifact. Confidence intervals remain wide due to the small sample size and should be interpreted accordingly.

#### 3.9.3. Decision Curve Analysis

**Figure 7:**
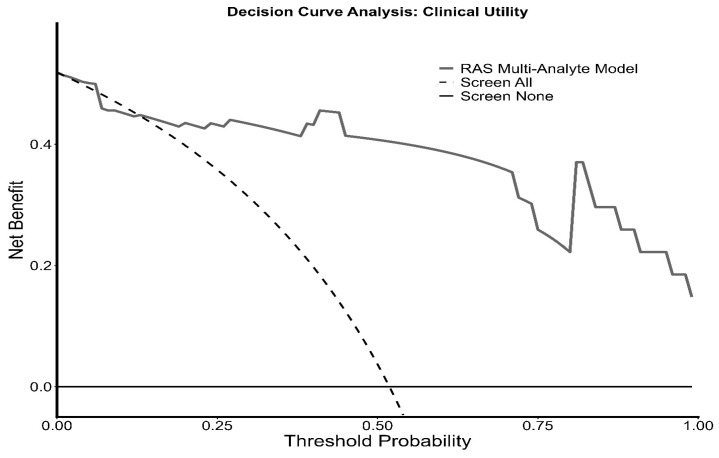
The Decision Curve shows that the RAS Multi-Analyte Model (bold grey line) maintains a superior net benefit over the ‘Screen All’ strategy (dashed line) across most threshold probabilities, particularly in the 20% to 50% risk range.

To assess the clinical net benefit of the RAS biomarkers, decision curve analysis (DCA) was employed. The model consistently outperformed the ‘screen all’ strategy across the majority of the threshold probability range. At a clinical threshold of 30%, the model yielded a net benefit of 0.43, compared to 0.31 for the ‘screen all’ approach. At a higher risk threshold of 50%, the model maintained a substantial net benefit (0.41) while the ‘screen all’ strategy offered no benefit (0.04) (Figure 7, Supplementary Material Table S5).

DCA is presented for exploratory purposes only. Given the small sample size of the study and the absence of external validation cohorts, these results do not support inferences about clinical utility, net clinical benefit, or reduction in unnecessary interventions in any target population. These findings should be considered illustrative and hypothesis-generating.

## 4. Discussion

In this study, we determined the concentrations of the RAS components (Ang 1-7, Ang II, Ang 1-7/Ang II, and ACE2) and a panel of cytokines (IL-6, IL-8, IL-10, and IFN-γ). We compared them between healthy controls and breast cancer patients to determine the effect of the disease on these biomarkers. Our results show that although Ang 1-7 and Ang II were not significantly affected by breast cancer, their ratio was significantly lowered. This finding indicates that breast cancer shifts the balance of RAS towards the pro-tumorigenic classical axis.

While the suppression of Ang 1-7 in breast cancer tissues is well-reported [21,43]. There was a significant gap in the circulating Ang 1-7 levels in breast cancer patients. In another study conducted in 102 breast cancer patients, serum Ang II level was identified as a strong predictor of breast cancer mortality [44]. Our results showed no alteration in circulating Ang 1-7 levels, and a small change in Ang II levels in breast cancer patients suggests that the absolute quantities of Ang 1-7 and Ang II alone may not be as informative in a small study population. Therefore, we normalized the concentration of Ang 1-7 with Ang II to obtain a ratio that could represent the RAS balance. We observed a significant reduction in Ang 1-7/Ang II, suggesting that the RAS balance shifted towards the classical axis. This finding is consistent with previous studies demonstrating dysregulation of the RAS in breast cancer tissues and cell lines [1,22,23,45].

Contrary to our findings, Zuo et al. found that plasma ACE2 was significantly lowered in breast cancer patients when compared to healthy controls. However, ACE2 concentrations were elevated after treatment with chemotherapeutic agents and were associated with a poor prognosis [46]. Our observation of elevated plasma ACE2 in treatment-naïve patients may appear contradictory, but several factors likely explain this discrepancy. First, the cancer stage in the two studies is substantially different: the former study included later-stage patients, while our cohort was predominantly early-stage (80.95% pT1). Temporal variation in ACE2 expression may reflect a compensatory early disease elevation that diminishes or reverses with progression or treatment. The other reason could be our cohorts being exclusively treatment-naïve, thereby removing the confounding effect of chemotherapy-induced ACE2 upregulation reported by Zuo et al. The third reason could be that racial and ethnic variability in ACE2 expression may contribute to differences across the study populations [47]. Elevated plasma ACE2 may also reflect ADAM17-mediated ectodomain shedding driven by tumor-associated inflammation or endothelial dysfunction, as observed in cardiovascular disease and COVID-19 [48,49,50]. Additionally, elevated circulating ACE2 protein levels do not necessarily reflect increased enzymatic activity, as ACE2 autoantibodies can neutralize catalytic activity [15,51]. The cardiometabolic comorbidities prevalent in our patient cohort (hypertension: 47.62%, cardiac arrhythmia: 19.05%) may also independently contribute to elevated circulating ACE2 [15,16]. Although sensitivity analysis restricted to non-hypertensive patients preserved the ACE2 elevation signal, the subgroup was very small (*n* = 6), and definitive attribution requires a larger, comorbidity-controlled study. These interpretations are speculative and require prospective mechanistic validation.

The role of IL-10 in breast cancer is not fully understood, although it is well accepted that IL-10 plays an important role. It acts as both a pro- and an anti-inflammatory cytokine, promoting and suppressing tumor progression [52,53]. IL-10 promotes tumor progression by activating STAT3, thereby inhibiting apoptosis and evading immune surveillance. Furthermore, it inhibits dendritic cell function, downregulates the human leukocyte antigen class I molecule on tumor cells, and suppresses the cytotoxicity of natural killer cells [54,55,56]. On the other hand, it inhibits tumor progression by downregulating the synthesis of pro-angiogenic factors and suppressing local pro-inflammatory and pro-tumorigenic cytokine release [28,57,58,59]. We observed lower IL-10 levels in breast cancer patients than in controls (Figure 3a). While IL-10 levels are generally shown to be elevated in cancer patients, studies have shown that plasma IL-10 levels can be lower in ER^+^ and PR^+^ breast cancer subtypes [60]. In addition, IL-10 levels were lowered after chemotherapy in ovarian cancer patients [61]. We also saw IL-10 levels significantly elevated in the hypertensive group. While the dysregulation of the IL-10 response is complex, IL-10 levels are expected to be lowered in chronic cardiovascular disease, such as hypertension [62]. Studies have shown that IL-10 levels can increase with antihypertensive therapy [63,64,65]. We also saw an increase in IL-10 levels in patients who used tobacco, which is consistent with previous studies [66,67,68]. While our findings are consistent with some previous findings, a study with a larger sample size considering a greater number of variables, such as use of antihypertensive drugs, duration of use, type of tobacco use (smoking, chewing, patch, etc.), status and duration of tobacco use, etc., is required to get a better picture of dysregulation of ACE2 and IL-10.

Although IL-8 was not measured as a primary outcome, and most samples fell below the LOQ, a non-significant trend towards higher plasma IL-8 levels in healthy controls than in breast cancer patients was observed. This is unexpected, given the well-established pro-tumorigenic and pro-angiogenic role of IL-8 in breast cancer [69]. The first possible explanation is the high number of below-LOQ values in both groups, which severely limits statistical power and introduces measurement bias. The other reason could be unmeasured inflammatory comorbidities or medications in the commercially sourced control group that could elevate baseline IL-8 levels. Shichkin et al. observed a similar trend in IL-8 levels in plasma and urine samples from breast cancer patients. This could presumably be due to increased clearance of inflammatory cytokines from the breast cancer tissue being shed into the bloodstream [70]. Additionally, the known effects of tobacco use (reported in 47.62% of patients) on the cytokine profiles. While tobacco generally increases pro-inflammatory cytokines, its net effect on IL-8 is context-dependent. This observation should not be over-interpreted and warrants prospective investigation in a larger, well-characterized cohort with matched controls.

We tested the association among the RAS components in all participants by calculating correlation coefficients. We did not see any correlation between Ang 1-7 and Ang II levels. This is consistent with a previous study showing there was no correlation between Ang 1-7 and Ang II in females [33], as our study cohort was exclusively composed of female subjects. Similarly, we did not observe a significant correlation between Ang 1-7 and Ang II individually, with ACE2, but we observed a moderate negative correlation between the Ang 1-7/Ang II ratio and ACE2. Although no direct correlations between ACE2 and the effector peptides have been previously evaluated, evidence indicates that plasma ACE2 is derived from ACE2 shedding from tissue-bound protein [71]. The soluble ACE2 retains its catalytic activity to degrade Ang II [72]. Therefore, with increased Ang II, plasma ACE2 may increase as a compensatory mechanism, leading to a negative correlation between ACE2 and the Ang 1-7/Ang II ratio [9].

We also saw that Ang II was negatively correlated with BMI. Under normal physiological conditions, adipose tissue expresses components of the RAS, such as angiotensinogen. Higher BMI, or more adipose tissue, means high angiotensinogen and subsequently high Ang II levels, leading to increased cancer risk. Many studies report that this holds across various pathological conditions, such as diabetes and cancer [18,73]. Interestingly, in a large study carried out in 2020, this trend was not true for females with breast cancer and cervical cancer. An inverse association for breast cancer incidence was seen when assessed against an increase in BMI [74]. Our findings support this notion, as we observe an inverse relationship between Ang II and BMI. This could be due to lower concentrations of estradiol and progesterone in the elderly females, low insulin-like growth factor-1 (IGF-1) levels and early differentiation of breast cancer tissues [74,75]. Additionally, the use of cytotoxic chemotherapy is often linked to cachexia or adipose tissue atrophy. This factor, combined with inflammation-induced high Ang II levels, may allow low BMI to coexist with high Ang II [76].

We saw a significant negative correlation between Ang 1-7 and platelets. Platelets express the Mas receptor and, upon activation, release nitric oxide, contributing to the antithrombotic effect [77]. Ang 1-7 has also been shown to stimulate platelet recovery in thrombocytopenia, suggesting a supportive role in platelet regeneration and production [78]. Hemoglobin did not show a significant correlation with Ang 1-7; however, it was positively correlated with Ang II and negatively correlated with the Ang 1-7/Ang II ratio. Ang II, via AT1R activation, stimulates erythropoietin secretion and acts as a growth factor for erythrocyte precursors in the bone marrow [79,80]. Treatments such as angiotensin receptor blockers (ARBs) and ACE inhibitors reduce hemoglobin and erythropoietin levels [80,81]. This shows that the contrary is consistent with the previous studies.

We determined an optimal model with a panel of biomarkers involving ACE2, Ang II and Ang 1-7 to predict the occurrence of breast cancer with excellent predictive performance using the Youden index, which offers the maximum potential effectiveness of a biomarker and is a common summary measure of the ROC curve. Although ACE2 alone showed strong discrimination between the groups, adding Ang 1-7 and Ang II to the model improved both the AUC and the model’s accuracy. The model, initially built with a standard cutoff of 0.5, also showed strong discrimination, with a Youden’s index of 0.86 and an optimal cutoff of 0.4203 for our combined model, indicating higher accuracy. The lower cutoff value may be due to the wide confidence intervals in the forest plot, suggesting high data variability. Although the model fared well at higher cutoffs, we optimized it to avoid misclassifications of patients as controls. The improvement in accuracy demonstrates meaningful clinical benefit from a combination of biomarkers, consistent with established findings that multiple biomarkers are better predictors than any single biomarker alone [82,83,84].

The forest plot (Figure 6) shows a detailed insight into the individual contributions of the biomarkers in the model. ACE2 was identified as the strongest independent predictor, with statistical significance, highlighting its central role in disease progression, consistent with previous studies showing that ACE2 may be altered during tumorigenesis and serve as a biomarker for multiple diseases [85,86,87,88,89]. Although statistically insignificant, Ang 1-7 showed a near-neutral effect and improved the model’s performance. This finding reflects the complex counter-regulatory balance of the protective RAS towards its negative impact. Ang II showed a substantially large positive association, highlighting its important role as an independent risk factor of breast cancer. This supports the fact that Ang II plays a critical role in cancer progression [45,90,91]. The overall model performance supports the clinical relevance of this comprehensive biomarker panel for cancer prediction, although further validation is needed to confirm its clinical utility.

### 4.1. Sample Size and Future Study Design

Post hoc power analysis (G*Power 3.1.9.7) indicated that group comparisons for Ang 1-7 (achieved power = 0.40), Ang II (0.55), Ang 1-7/Ang II ratio (0.73), and IL-10 (0.50) were underpowered. The ACE2 group comparison achieved a reasonable pilot-study-level power of 0.94. To achieve a power greater than 0.95 at a two-sided α = 0.05, future studies would require approximately 89 participants per group for Ang 1-7, 49 for Ang II, 34 for the ratio, and 66 for IL-10. Future studies should also stratify participants by molecular subtype (ER/PR/HER2 status), tumor stage, and grade to evaluate whether circulating RAS imbalance differs across biologically distinct breast cancer subtypes. These estimates are provided to inform the design of future confirmatory studies and are not intended as a post hoc justification of the current findings.

### 4.2. Limitations

This study has several important limitations that must be acknowledged. First, the sample size was small (17 controls and 21 patients), and the complete-case multivariate model included only 27 participants. This substantially limits statistical power, increases the risk of overfitting, and constrains the stability of logistic regression estimates. This is reflected in the wide confidence intervals for ACE2 and Ang II odds ratios. Furthermore, patient samples were sourced from St. Luke’s Hospital, while control samples were obtained commercially (SpeciCare). Only age and sex were available for the control cohort. This can potentially lead to pre-analytical differences in sample handling, storage duration, and population-level unmeasured comorbidities between sources that cannot be fully excluded. The high prevalence of hypertension (47.62%), tobacco use (47.62%), and cardiovascular comorbidities in patients, with limited clinical characterization of the control group, prevents definitive attribution of the observed RAS imbalance to breast cancer biology alone. Moreover, exclusion of samples due to hemolysis and values below the limit of quantification may have introduced measurement bias; participants whose samples were excluded from specific analytes may differ systematically from those retained.

Additionally, the cohort is predominantly early-stage (80.95% pT1) and hormone receptor-positive (ER+: 85.71%; PgR+: 80.95%), limiting generalizability to other breast cancer subtypes. The cross-sectional design also does not permit causal inference or assessment of prognostic value. External validation in an independent, larger, prospectively enrolled cohort with matched controls is essential before the proposed biomarker panel can be considered for any clinical application.

## 5. Conclusions

In this small pilot case–control study, we observed elevated ACE2 levels, a reduced Ang 1-7/Ang II ratio, and decreased IL-10 levels in treatment-naïve breast cancer patients compared with healthy controls. A multi-analyte regression model combining ACE2, Ang 1-7 and Ang II demonstrated promising exploratory discriminatory performance. These findings are consistent with the hypothesized shift towards the pro-tumorigenic classical RAS axis in breast cancer, with altered immune modulation. These results should be interpreted with caution. The multivariate model was fitted to only 27 participants, raising concerns about overfitting that internal bootstrap validation alone cannot address. These case–control samples were sourced from different institutions, which limits the ability to exclude pre-analytical confounding. The high prevalence of hypertension (47.62%) and other cardiometabolic comorbidities in the patient cohort may have independently influenced circulating RAS components. The cohort was predominantly early-stage and hormone receptor-positive, limiting generalizability to other breast cancer subtypes. These results are hypothesis-generating and should be considered a foundation for future work. Prospective studies with prospectively enrolled, comorbidity-matched controls, larger sample sizes, subtype stratification, and longitudinal outcome data are required to confirm the discriminatory performance and investigate the biological and clinical significance of circulating RAS imbalance in breast cancer.

## Figures and Tables

**Table 1:** Clinical characteristics, comorbidities, disease state, and medication history of breast cancer patients.

Clinical Characteristics	
Age (years)	65.14 ± 12.98
BMI (kg/m^2^)	33.44 ± 14.60
Pathological tumor and nodal classification (pTNM)	*n* (%)
pT1a	4 (19.05)
pT1b	4 (19.05)
pT1c	9 (42.85)
pT2	3 (14.29)
pN0	16 (76.19)
pN1a	2 (9.52)
pN1c	1 (4.76)
pN1mi	1 (4.76)
Tumor grade	*n* (%)
G1	16 (76.19)
G2	3 (14.29)
G3	1 (4.76)
ER+, *n* (%)	18 (85.71)
PgR+, *n* (%)	17 (80.95)
HER2+, *n* (%)	4 (19.05)
Comorbidities	*n* (%)
Diabetes	2 (9.52)
Cardiac arrhythmia	4 (19.05)
Hypertension	10 (47.62)
Peripheral Vascular Disease	1 (4.76)
Congestive Heart Failure	1 (4.76)
Tobacco use, *n* (%)	10 (47.62)
Medication History *	*n* (%)
Aspirin (any dose)	3 (14.29)
NSAIDs	4 (19.05)
Statins	4 (19.05)
Platelets (per µL)	(255.74 ± 55.4) × 10^3^
Total Bilirubin (mg/dL)	0.65 ± 0.40
Creatinine (mg/dL)	0.79 ± 0.18
eGFR (mL/min/1.73 m^2^)	84.17 ± 20.70
WBC (per µL)	(7.32 ± 2.20) × 10^3^
Hemoglobin (g/dL)	13.02 ± 1.66

Data presented as Mean ± SD unless stated otherwise. BMI, Body Mass Index; HER2+, Human Epidermal Growth Factor Receptor 2 positive; NSAID, non-steroidal Anti-inflammatory drugs; eGFR: Estimated Glomerular Filtration Rate (CKD-EPI 2021 equation); WBC, white blood cells. N = 20 for all variables except Age, where N = 21. Pathological staging follows AJCC 8th edition TNM criteria; all patients were pM0 based on clinical assessment at the time of sample collection. Clinical stage is based on the standard pTNM classification, where T = tumor, N = nodes, and M = metastasis. Tumor grades are classified based on differentiation and aggressiveness, where G1 = well differentiated and least aggressive; G2 = moderately differentiated and moderately aggressive, and G3 = poorly differentiated and most aggressive. Control group laboratory and medication data were unavailable because the sample source was a commercial vendor (SpeciCare). * Specific antihypertensive class data were not available for all patients; this is acknowledged as a limitation.

## Data Availability

The datasets used and analyzed during the current study are available from the corresponding author on reasonable request.

## Supplementary Materials

The following supporting information can be downloaded at: https://www.mdpi.com/article/10.3390/biomedicines14051086/s1, Table S1. Group comparison of different analytes between control group and patients. Table S2. The correlation of RAS components with different variables in control and patient groups. Table S3. The Correlation of ACE2 and IL-10 with different variables in control and patient groups. Table S4. The coefficients for Firth’s penalized logistic regression for combined ACE2, Ang 1-7 and Ang II model. Table S5. Comparison of Clinical Net Benefit Between the RAS Multi-Analyte Model and Standard Screening Strategies Across Various Threshold Probabilities.

## Abbreviations

The following abbreviations are used in this manuscript:

RAS	Renin Angiotensin System
ACE	Angiotensin-Converting Enzyme
Ang I	Angiotensin I
Ang II	Angiotensin II
Ang 1-7	Angiotensin 1-7
AT1R	Angiotensin II Type 1 Receptor
AT2R	Angiotensin II Type 2 Receptor
MasR	Mas Receptor
NF-κB	Nuclear factor kappa B
FAK	Focal adhesion kinase
EMT	Epithelial–mesenchymal transition
SPE	Solid Phase Extraction
MRM	Multiple reaction monitoring
ESI	Electrospray ionization
CE	Collision energy
DP	Declustering potential
CXP	Cell exit potential
BMI	Body mass index
HER2^+^	Human Epidermal Growth Factor Receptor 2 positive
NSAIDs	Non-steroidal anti-inflammatory drugs
T	Tumor
N	Nodes
M	Metastasis
pN	Pathological N categories
ER^+^	Estrogen receptor positive
PgR^+^	Progesterone receptor positive
AIC	Akaike Information Criterion
R^2^_McF_	McFadden’s pseudo R^2^
VIF	Variance inflation factor
ROC	Receiver operating characteristic
