# Supplementary material for: Exploratory Analysis of Plasma Angiotensin-Converting Enzyme 2 and Angiotensin Peptides as Candidate Discriminatory Signals in Breast Cancer: A Pilot Case–Control Study
Source: Biomedicines. 2026 May 12;14(5):1086. doi: 10.3390/biomedicines14051086 (PMC13204482; doi:10.3390/biomedicines14051086)
Supplement: Supplementary file 1 [file biomedicines-14-01086-s001.zip › biomedicines-4285194-supplementary.pdf]

Table S1. Group comparison of different analytes between control group and patients								
**Analyte (conc.)**	**Control Avg±SEM**	**Patients Avg±SEM**	**P-value**	**Rank biserial correlation (r)/Effect size (D)**	**95% CI**	**Post-hoc Power**	**Control (N)**	**Patients (N)**
**Ang 1-7 (ng/ml)**	43.40±13.04	24.39±2.23	0.5814	-0.1177	NA	0.400	16	17
**Ang II (ng/ml)**	0.12 ±0.01	0.20±0.04	0.3591	0.2048	NA	0.546	14	15
**Ang 1-7/ Ang II**	473.20±129.07	169.47±30.33	**0.0442**	-0.4196	NA	0.726	15	17
**ACE2 (ng/ml)**	0.74±0.04	1.33±0.17	**0.0005**	0.6678	NA	0.939	16	19
**IL-10 (pg/ml)**	1.53±0.09	1.28±0.08	**0.0420**	0.7302	0.0096-0.4896	0.501	15	19
**IL-6 (pg/ml)**	8.58 ± 2.42	30.45 ± 18.68	0.2883	NA	NA	NA	10	7
**IL-8 (pg/ml)**	3.75 ±1.48	1.73 ± 0.57	0.2229	NA	NA	NA	12	7
**IFN-γ (pg/ml)**	12.51 ± 2.23	38.87 ± 25.33	0.3468	NA	NA	NA	5	6

Data are presented as Mean ± SEM and expressed in ng/mL for RAS components and pg/mL for cytokines. RAS components were non-normally distributed and analyzed using nonparametric Mann Whitney U test by calculating rank biserial correlation (r) and respective p values to assess statistical significance. IL-10 was compared using student's t-test with welch correction for variance due to normally distributed data. Effect size (D), p-value and 95% Confidence Interval (CI) were calculated to assess the statistical significance. IL-6, IL-8, and IFN-γ were not included in group comparison due to less than 50% of the samples from at least one group being below limit of quantification. Post hoc power calculation was carried out to identify the minimum number of required samples for future studies.

Abbreviations: Ang 1-7 = Angiotensin 1-7; Ang II = Angiotensin II; ACE2 = Angiotensin-converting enzyme 2; IL = Interleukin; IFN-γ = Interferon-γ

Exploratory Subgroup Analyses (Hypothesis-Generating Only; n per subgroup ≤ 10)

As there was a significant difference in IL-10 levels between groups, we analyzed the effects of hypertension on components of the RAS and IL-10. We did not observe a significant difference (r = 0.0204, p = 1.0000) in Ang 1-7 levels between the non-hypertensive (26.09 ± 4.50, n = 7) and hypertensive groups (23.92 ± 2.42, n = 7). Similarly, no differences were observed in Ang II levels (r = -0.0952, p = 0.8357, n = 6, 7) or in Ang 1-7/Ang II levels (r = -0.1111, p = 0.7756, n = 6, 9) between hypertensive and non-hypertensive groups. ACE2 (r = -0.7778, p = 0.0120, n = 6, 9) was significantly reduced in the hypertensive group, whereas IL-10 (D = -1.0578, p = 0.0488, n = 6, 10) was significantly elevated. Similarly, the use of tobacco did not have significant effect in the Ang 1-7 (r = 0.3016, p = 0.3511), Ang II (r = 0.2143, p = 0.5358), Ang 1-7/Ang II (r = -0.0833, p = 0.8148), and ACE2 (r = -0.2099, p = 0.4894) levels (Fig. 4b). IL-10 was significantly elevated (D = -1.1345, p = 0.0270) among tobacco users. To further assess the potential confounding effect of hypertension on primary study outcomes, a sensitivity analysis was performed by restricting the

cancer group to non-hypertensive patients and comparing them against the full control cohort. ACE2 remained significantly elevated in non-hypertensive cancer patients (1.65 ± 0.62 ng/mL) compared to controls (0.74 ± 0.04 ng/mL) ($p < 0.0001$), reinforcing that the ACE2 elevation observed in the primary analysis reflects cancer-associated biology rather than hypertension related confounding. In contrast, the Ang 1-7/Ang II ratio did not reach statistical significance in this restricted sub-group ($p = 0.3403$), likely attributable to the reduced statistical power resulting from the smaller cancer sub-group ($n = 6$). The directional trend remained consistent with the primary analysis (188.40 ± 38.48 in cancer vs. 473.20 ± 129.07 in controls), suggesting that the loss of significance reflects insufficient power rather than the absence of effect.

Table S2. The correlation of RAS components with different variables in control and patient groups.

Variables	Ang 1-7			Ang II			Ang 1-7/Ang II		
	r	p-value	N	r	p-value	N	r	p-value	N
**Ang 1-7**			33	0.1641	0.3950	29	0.3764	0.0442	29
**Ang II**	0.1641	0.3950	29			29	-0.7859	0.0000	29
**Ratio**	0.3764	0.0442	29	-0.7859	0.0000	29			32
**ACE2**	-0.3322	0.0729	30	0.1811	0.3659	27	-0.3218	0.0829	30
**IL-10**	0.3236	0.0811	30	-0.1092	0.5954	26	0.1518	0.4406	28
**Age**	0.0982	0.5865	33	0.2076	0.2798	29	-0.2611	0.1489	32
**BMI**	-0.0676	0.8051	16	**-0.5516**	**0.0438**	**15**	0.2426	0.3467	17
**Platelet**	**-0.5147**	**0.0436**	**16**	0.1750	0.5320	15	-0.3995	0.1132	17
**Bilirubin**	0.3800	0.1797	14	0.3329	0.2647	13	-0.0797	0.7772	15
**Creatinine**	0.3545	0.1772	16	0.2883	0.2950	15	-0.0994	0.7025	17
**WBC**	-0.2735	0.3043	16	0.2536	0.3607	15	-0.2647	0.3034	17
**Hemoglobin**	-0.1193	0.6579	16	**0.5827**	**0.0248**	**15**	**-0.6270**	**0.0083**	**17**

Bold p-values indicate statistical significance ($p < 0.05$). r = Spearman's rho, N = sample size. Abbreviations: Ang 1-7 = Angiotensin 1-7; Ang II = Angiotensin II; ACE2 = Angiotensin-converting enzyme 2; IL-10 = Interleukin 10; BMI = Body Mass Index; WBC = White Blood Cells; Ratio = Ang 1-7/Ang II.

Table S3. The Correlation of ACE2 and IL-10 with different variables in control and patient groups

Variables	ACE2			IL-10		
	r	p-value	N	r	p-value	N
**Ang 1-7**	-0.3322	0.0729	30	0.3236	0.0811	30
**Ang II**	0.1811	0.3659	27	-0.1092	0.5954	26
**Ratio**	-0.3218	0.0829	30	0.1518	0.4406	28
**ACE2**	-	-	-	**-0.4123**	**0.0212**	**31**
**IL-10**	**-0.4123**	**0.0212**	**31**	-	-	-
**Age**	0.0960	0.5833	35	0.0449	0.8009	34
**BMI**	0.1084	0.6687	18	0.2043	0.4161	18
**Platelet**	0.0221	0.9359	17	-0.2312	0.3688	17

**Bilirubin**	-0.1736	0.5339	15	0.4165	0.1227	15
**Creatinine**	0.2947	0.2491	17	-0.0967	0.7093	17
**WBC**	**0.5515**	**0.0237**	17	-0.0311	0.9064	17
**Hemoglobin**	-0.2663	0.2993	17	0.2316	0.3673	17

Bold p-values indicate statistical significance ($p < 0.05$). r = Spearman's rho, N = sample size.
Abbreviations: Ang 1-7 = Angiotensin 1-7; Ang II = Angiotensin II; ACE2 = Angiotensin-converting enzyme 2; IL-10 = Interleukin 10; BMI = Body Mass Index; WBC = White Blood Cells; Ratio = Ang 1-7/Ang II.

Table S4. The coefficients for Firth's penalized logistic regression for combined ACE2, Ang 1-7 and Ang II model.

Predictor	Coefficient	OR	95% CI	p-value
**Intercept**	-7.486	0.0006	9.48×10^{-8} – 0.10	0.0011
**ACE2**	7.405	1644.86	10.29 – 7,911,000	0.0004
**Ang 1-7**	0.0001	1.00	0.9674 – 1.0190	0.9902
**Ang II**	3.905	49.63	0.4412 – 4.28×10^{6}	0.2710

Table S5. Comparison of Clinical Net Benefit Between the RAS Multi-Analyte Model and Standard Screening Strategies Across Various Threshold Probabilities.

Threshold Probability (Pt)*	RAS Multi-Analyte Model (Net Benefit)	Screen All Patients (Net Benefit)	Screen None (Net Benefit)
0.10 (10%)	0.4527	0.465	0
0.20 (20%)	0.4352	0.3981	0
0.30 (30%)	0.4339	0.3122	0
0.40 (40%)	0.4321	0.1975	0
0.50 (50%)	0.4074	0.037	0
0.60 (60%)	0.2037	-0.2037	0

Decision Curve Analysis (DCA) compares the clinical utility of the RAS Multi-Analyte Model against two baseline strategies: treating all patients ("Screen All") or treating none ("Screen None"). The 'Screen None' strategy is set at 0.0 to serve as the reference baseline, representing the net clinical utility when no intervention is applied.
RAS Multi-Analyte Model (Net Benefit): Higher values indicate superior clinical utility.
The RAS Multi-Analyte Model consistently maintains a positive net benefit across the threshold probability range (P_t = 0.20 to 0.60). Notably, at a 50% threshold, the model provides a substantial net benefit (0.41) while the "Screen All" strategy drops to near-zero (0.04), indicating the model effectively reduces over-intervention without sacrificing clinical gain.
The negative value for "Screen All" at 0.60 (-0.2037) indicates that the harm of unnecessary screening outweighs the benefit at that high-risk threshold.
